# The suitability of *NONO-TFE3* dual-fusion FISH assay as a diagnostic tool for *NONO-TFE3* renal cell carcinoma

**DOI:** 10.1038/s41598-020-73309-4

**Published:** 2020-10-01

**Authors:** Ning Liu, Wei Guo, Qiancheng Shi, Wenyuan Zhuang, Xiaohong Pu, Shaoyu Chen, Feng Qu, Linfeng Xu, Xiaozhi Zhao, Xiaogong Li, Gutian Zhang, Hongqian Guo, Weidong Gan, Dongmei Li

**Affiliations:** 1 Department of Urology, Nanjing Drum Tower Hospital, The Affiliated Hospital of Nanjing University Medical School, 321 Zhongshan Road, Nanjing, 210008 Jiangsu China; 2 Department of Urology, Drum Tower Clinical Medical School of Nanjing Medical University, Nanjing, Jiangsu China; 3 Department of Urology, Jiangsu Taizhou People’s Hospital, Taizhou, Jiangsu China; 4 Department of Pathology, Nanjing Drum Tower Hospital, The Affiliated Hospital of Nanjing University Medical School, Nanjing, Jiangsu China; 5 Guangzhou LBP Medicine Science & Technology Co., LTD, Guangzhou, Guangdong China; 6 Immunology and Reproduction Biology Laboratory & State Key Laboratory of Analytical Chemistry for Life Science, Medical School, Nanjing University, 22 Hankou Road, Nanjing, 210093 Jiangsu China; 7 Jiangsu Key Laboratory of Molecular Medicine, Nanjing University, Nanjing, Jiangsu China

**Keywords:** Renal cell carcinoma, Cancer of unknown primary, Cancer genetics

## Abstract

*NONO-TFE3* RCC is a subtype of Xp11.2 translocation renal cell carcinoma (RCC). So far, only a small amount of *NONO-TFE3* RCC have been reported owing to lack of effective diagnosis methods. Utilizing the novel dual-fusion fluorescence in situ hybridization (FISH) probe reported here, 5 cases of *NONO-TFE3* RCC were identified and were ultimately confirmed by RT-PCR. Histopathology, all 5 cases were consisted by sheets of epithelial cells and papillary architecture. The cytoplasm was abundantly clear, and nucleoli was not prominent. Besides, the nuclear palisading, subnuclear vacuoles and psammoma bodies were identified. The most distinctive features were strong positive TFE3 staining but equivocal split signals of the *TFE3* probe, which might lead to the misdiagnosis of Xp11.2 translocation RCC. The median age and median tumor size of the five patients were 41.2 years and 3.6 cm, respectively. A median following follow-up of 27 months showed moderate disease progression and prognosis in *NONO-TFE3* RCC patients. In conclusion, the present study demonstrates the effectiveness and reliability of the *NONO-TFE3* dual-fusion FISH probe for diagnosing *NONO-TFE3* RCC. Suspected cases of Xp11.2 translocation RCC showing biphasic pattern, strong positive TFE3 staining, and equivocal split signals in the TFE3 FISH assay indicated a possibility of *NONO-TFE3* RCC.

## Introduction

Renal cell carcinoma (RCC) associated with Xp11.2 translocation/*TFE3* gene fusion (Xp11.2 translocation RCC) is a rare but aggressive type of renal tumor. In 2016^1^, Xp11.2 translocation RCC was identified as translocation RCC of the microphthalmia (MiTF) family of transcription factors. According to the fusion pattern, Xp11.2 translocation RCC can be divided into at least 10 subtypes, and tumors with different specific gene fusions are associated with distinctive biological processes^2–4^. For instance, *ASPSCR1-TFE3* RCC, one of the most common subtypes of Xp11.2 translocation RCC, was reported to be more malignant than other subtypes^5,6^. *NONO-TFE3* RCC, which involved in the inv(X) (p11.2; q12) chromosomal variation, accounts for 12.5% of the Xp11.2 translocation RCC patient population^7,8^. However, less than 20 cases of *NONO-TFE3* RCC have been reported since the first cases in 1997^9,10^. This is because most cases of *NONO-TFE3* RCC were remain undiagnosed or incorrectly diagnosed.

In general, TFE3 immunohistochemical (IHC) and TFE3 break-apart fluorescence in situ hybridization (FISH) assays are the most popular methods for diagnosing Xp11.2 translocation RCC^5,11^. Xp11.2 translocation RCC is typically characterized by strong nuclear immunoreactivity with TFE3 antibody and a larger split signal in the TFE3 break-apart FISH assay, respectively^12^. However, TFE3 immunoreactivity only reflects the expression levels of TFE3 protein, and the FISH split signal merely indicates rearrangements in *TFE3*. Identification of fusion patterns still relies on fusion FISH assays^5,13^, reverse transcription-polymerase chain reactions (RT-PCR), cytogenetic karyotypic analysis^10^, or sequencing^14^. However, the fusion FISH assay represents the most inexpensive and accessible method, due to economic difficulties and complicated sample requirements of the other methods.

*NONO* gene, a member of the *Drosophila* behavior/human splicing (DBHS) protein family, is located adjacent to *TFE3*. However, the adjacent location of *NONO* and *TFE3* genes result in an equivocal split signal distance of TFE3, which leads to the misdiagnosis of *NONO-TFE3* RCC as non-Xp11.2 translocation RCC^9,15^. Previously, Argani and Xia et al.^8,9^ introduced their single-fusion *NONO-TFE3* FISH assays. In the present study, we designed a novel *NONO-TFE3* dual-fusion FISH assay and identified five cases of *NONO-TFE3* RCC based on our FISH probe. Therefore, the present study was designed to determine the effectiveness of the novel assay and highlight the diagnostic potential of dual-fusion and single-fusion FISH assays.

## Results

### FISH analysis

**Figure 1:**
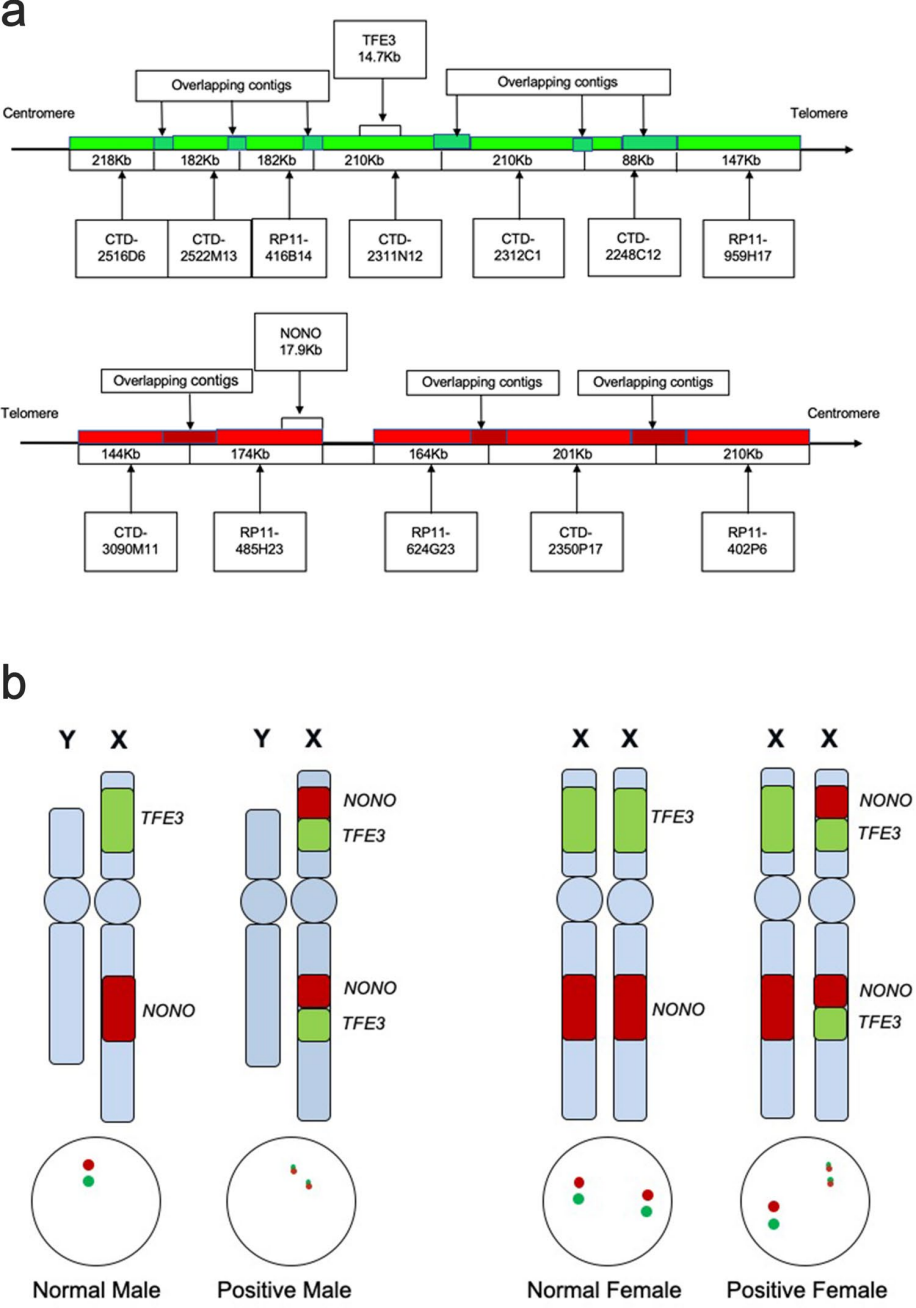
A schematic representation of the *NONO-TFE3* dual-fusion probe. (**a**) Bacterial artificial chromosomes (BACs) labeled with red fluorescence covered the almost entire *NONO* gene, and BACs labeled with green fluorescence covered the entire *TFE3* gene; (**b**) the principle of the *NONO-TFE3* dual-fusion FISH assay.

**Figure 2:**
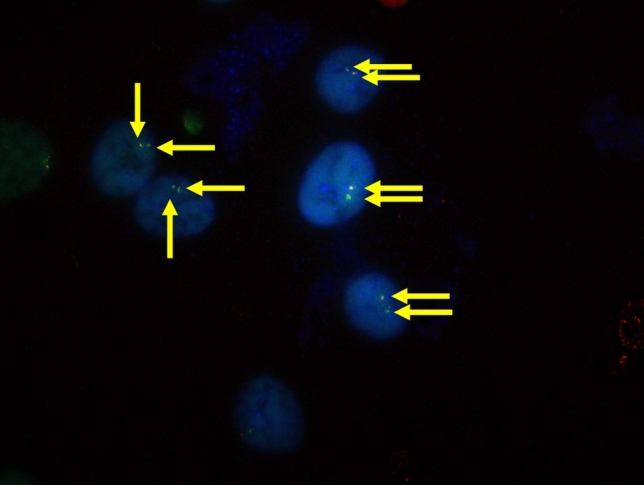
Typical positive results of *NONO-TFE3* dual-fusion FISH assay in UOK 109 cell (2F, yellow arrowheads).

As displayed in Fig. 1a, *NONO* gene was labeled with red fluorescence and *TFE3* gene was green fluorescence by bacterial artificial chromosomes (BACs). In general, signals were considered to be fused when yellow and abutting (equal to or less than the one signals’ diameters) green–red signals were observed. Given the small gap between gene specific probe (GSP) *TFE3* and GSP *NONO* (nearly 21 Mb), controls might also show adjacent or partially overlapping signals. Therefore, patient gender and signal sizes needed to be taken into consideration for the evaluation of FISH results (Fig. 1b). For males, when the 2 fusion signals (2F) appeared simultaneously, the reciprocal translocation between the *NONO* and *TFE3* genes could be confirmed (Fig. 2). For female cases, a pair of split signals (1G1R) were also required. In contrast, the presence of one fusion signal in males, and two fusion signals unaccompanied with two split signals in females, both represented adjacent *TFE3* and *NONO* signals. By this method, the observed fusion and split signals could be accurately identified as *NONO-TFE3* fusion genes. In addition, signal size was another factor used to identify the signal patterns. Fusion signals were consisted of two split signals, with sizes markedly smaller than integrated green or red signals. Therefore, there were two pairs of small split signals and one pair of large unsplit signal in females with the *NONO-TFE3* gene fusion.

**Figure 3:**
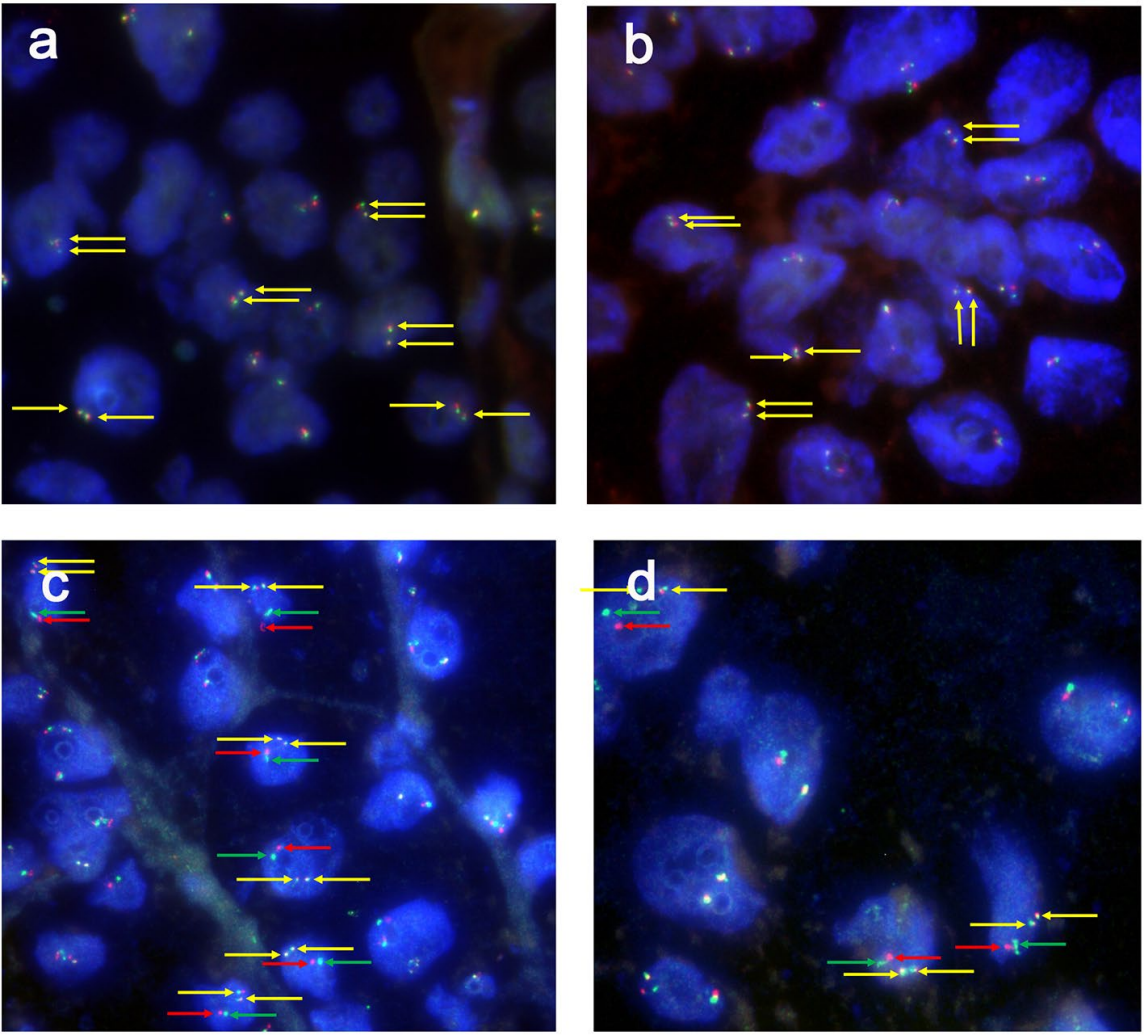
(**a**, **b**) show typical positive results of *NONO-TFE3* dual-fusion FISH assay in males (2F, yellow arrowheads); (**c**) and (**d**) show typical positive results of *NONO-TFE3* dual-fusion FISH assay in females (2F1G1R, yellow, red and green arrowheads).

**Figure 4:**
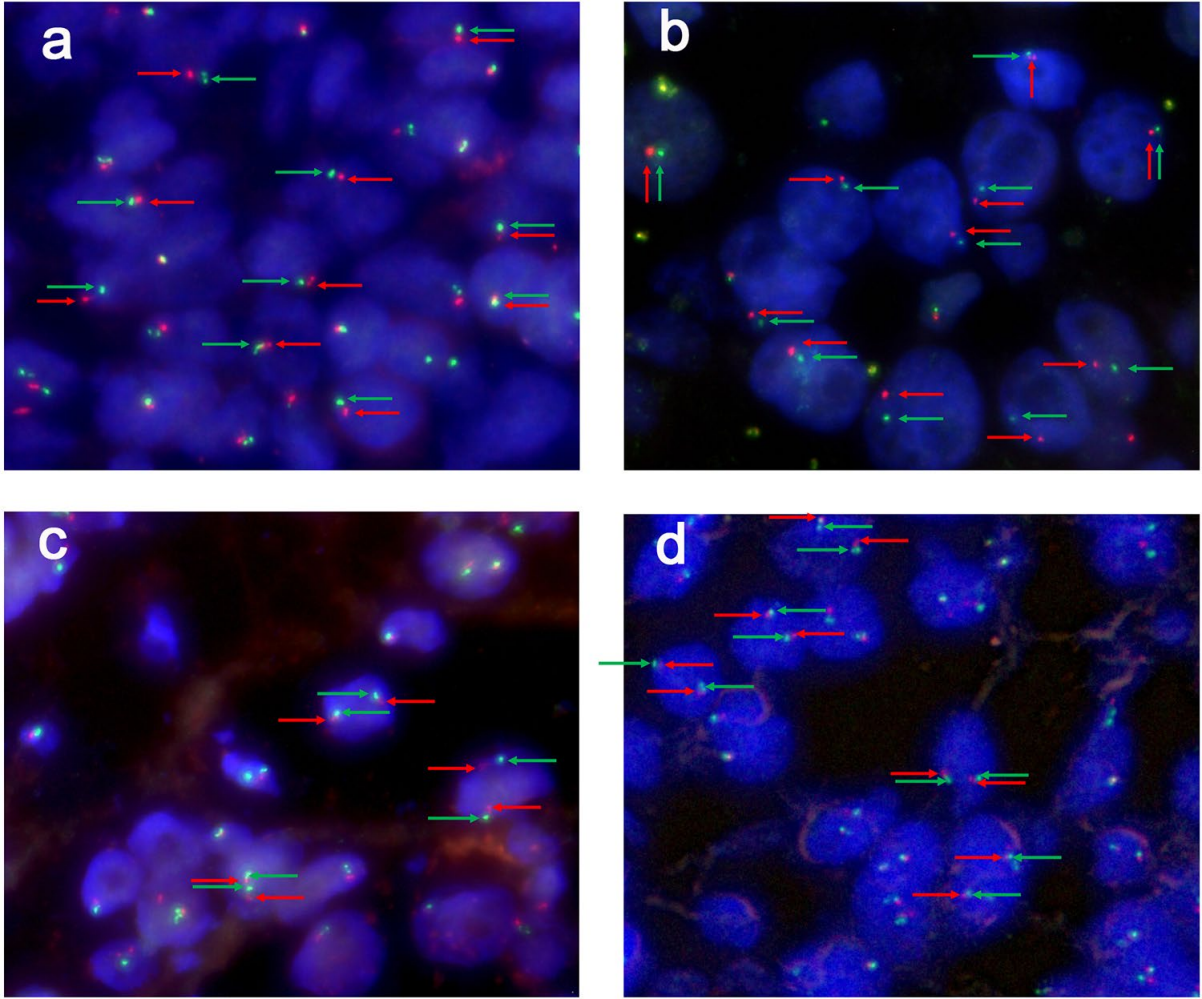
(**a**, **b**) show typical negative results of *NONO-TFE3* dual-fusion FISH assay in males (1G1R, red and green arrowheads); (**c**, **d**) show typical negative results of *NONO-TFE3* dual-fusion FISH assay in females (2G2R, red and green arrowheads).

In total, there were 5 cases were showed *NONO* gene and *TFE3* gene fusion. All five cases showed equivocal split signals with distances of nearly one signal diameters, which might be analyzed as a false-negative diagnosis by an unexperienced pathologist. For males with *NONO-TFE3* translocation (Fig. 3a,b), two fusion signals (2F) were observed in 14.1–35% of the tumor cells, and a pair of split signals as well as a fusion signal (1G1R) were observed in 13–25% cells. For *NONO-TFE3* dual-fusion probe positive females (Fig. 3c,d), two fusion signals in addition to a pair of split signals (1G1R2F) were observed in 8–21% cells, and two pairs of split signals (2G2R) were observed in 19–27% cells. For negative cases (Fig. 4), such as ccRCC and pRCC, 2F and 1G1R signal patterns were observed in 1% and 22–88.35% of male tumor cells, respectively. Moreover, 1G1R2F and 2G2R signal patterns were observed in 3% and 4.71–67.3% of female tumor cells. In total, the rate of false positive detection of this fusion FISH probe was 1.8%, and the threshold value was set to 2%.

**Figure 5:**
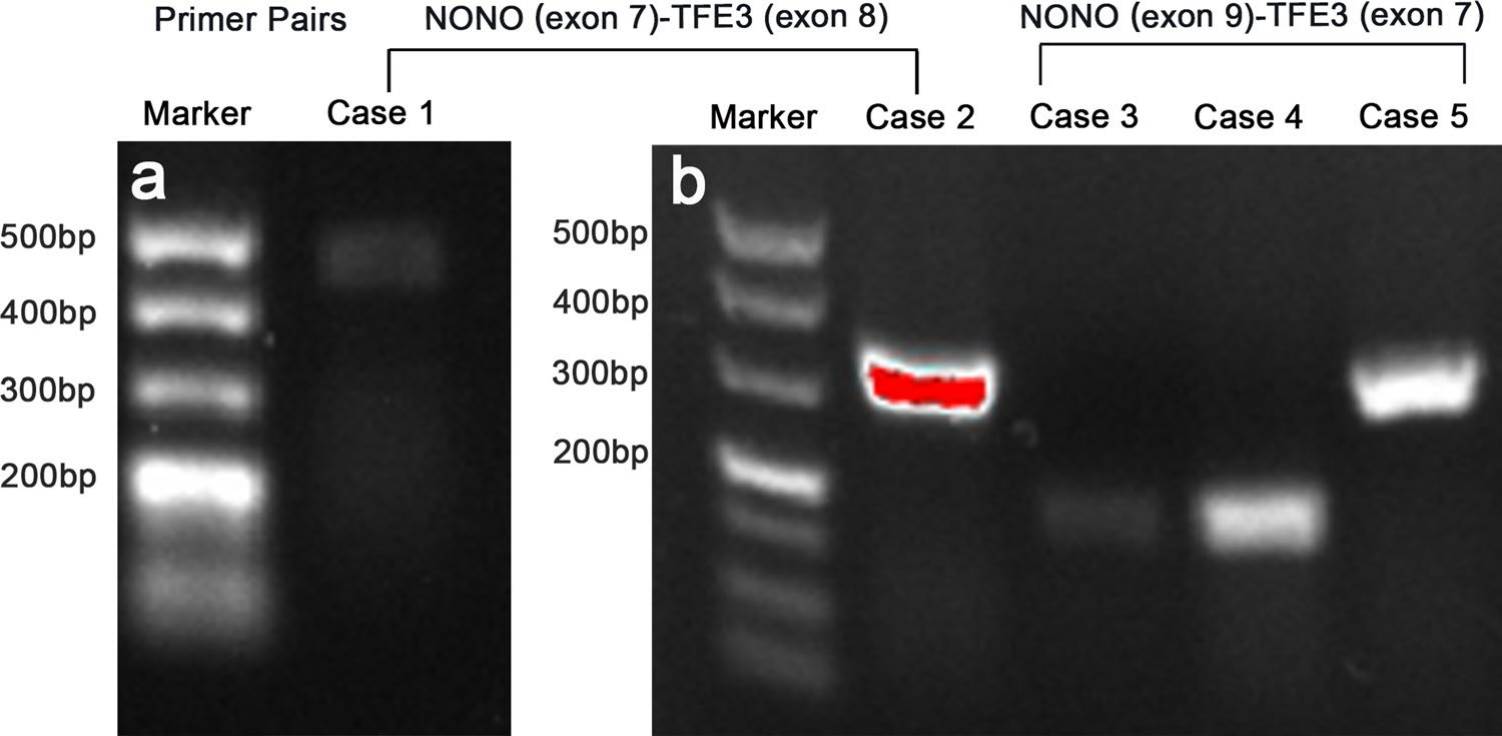
(**a**)The results of reverse transcriptase–PCR showed a 500 bp targeted *NONO-TFE3* transcript by the primer pairs *NONO* exon 7 (F) and *TFE3* exon 8 (R); (**b**) Case 2 showed a 300 bp targeted *NONO-TFE3* transcript by the primer pairs *NONO* exon 7 (F) and *TFE3* exon 8 (R), while 3, 4 and 5 showed respectively 200 bp and 500 bp targeted *NONO-TFE3* transcript by the primer pairs *NONO* exon 9 (F) and *TFE3* exon 7 (R).

**Figure 6:**
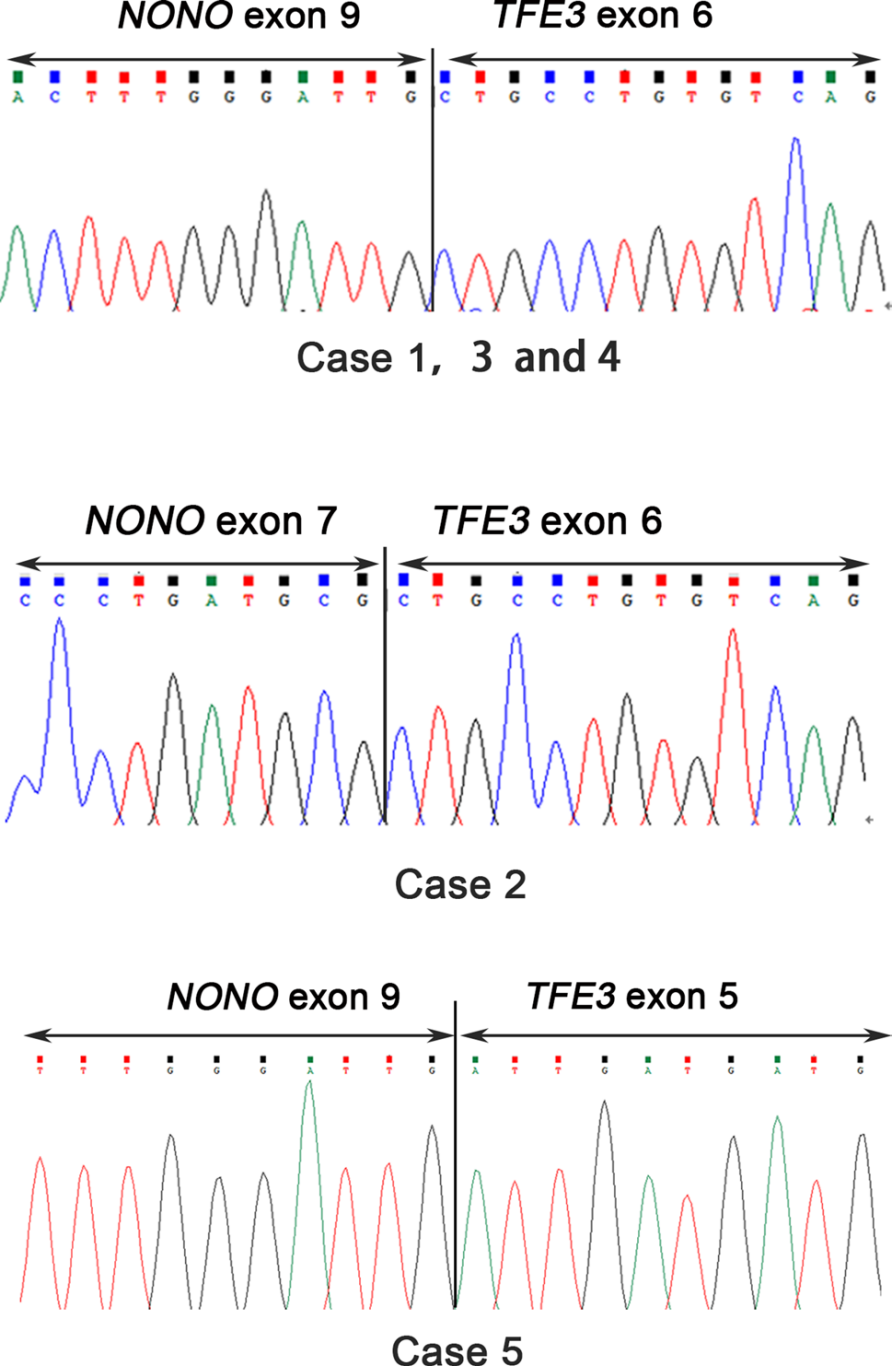
The *NONO-TFE3* fusion gene patterns among five cases: case 1, 3 and 4 had a fusion pattern between exon 9 of *NONO* and exon 6 of *TFE3*, cases 2 had a fusion pattern between exon 7 of *NONO* and exon 6 of *TFE3*, while case 5 had a fusion pattern between exon 9 of *NONO* and exon 5 of *TFE3*.

Adequate RNA was extracted from FFPE tissues from all 5 patients and subjected to RT-PCR analysis. ~ 200 bp bands extracted from two cases and a ~ 300 bp bands were reverse transcribed by the primer pairs *NONO* exon 9 (F) and *TFE3* exon 7 (R). On the other hand, a ~ 300 bp bands and a ~ 500bps band from the remaining patient were reverse transcribed by the primer pairs *NONO* exon 7 (F) and *TFE3* exon 8 (R). All PCR products were successfully extracted and further analyzed (Fig. 5). Finally, three fusion patterns were identified, including 3 cases (cases 1, 3 and 4) of a fusion point between exon 9 of *NONO* and exon 6 of *TFE3*, one case (cases 2) of a fusion point between exon 7 of *NONO* and exon 6 of *TFE3*, and one case (case 5) of a fusion point between exon 9 of *NONO* and exon 5 of *TFE3* (Fig. 6).

### Patients

Including 8 cases of *ASPSCR1-TFE3* RCC, 8 cases of *PRCC-TFE3* RCC and the 13 cases of fusion partner undefined Xp11.2 translocation RCC, *NONO-TFE3* RCC in our study accounted for 17.2% of the total population of Xp11.2 translocation RCC patients. The clinicopathological features of the 5 *NONO-TFE3* RCC patients are listed in Table 1. Of the 5 cases of *NONO-TFE3* RCC, 4 cases were initially diagnosed as Xp11.2 translocation RCC and the other one was negative to the TFE3 break-apart probe. The mean age of these patients was 41.2 years and the mean tumor size was 3.6 cm. Four patients were treated with nephron-sparing surgery (NSS), with radical nephrectomy (RN) conducted on the remaining patient diagnosed with a tumor near the pelvis. According to the 2010 American Joint Committee on Cancer TNM staging system, 4 of these patients were classified as stage I and one as stage III. Follow-up studies were conducted on all five patients from 5 to 51 months (mean, 28.2 months; median, 27 months). Four patients were found to be alive with no evidence of disease, but a 42-year-old man died 28 months after the surgery.

**Table 1:** Clinical data of the Xp11.2 translocation renal cell carcinoma.

Case	Fusion type	Age/sex	Tumor size (cm)	AJCC Stage	Treatment	Follow-up (month)
1	*NONO-TFE3*	55/F	3	pT3aNxM0(III)	RN	14, NED
2	*NONO-TFE3*	42/M	3.5	pT1aM0N0(I)	NSS	Peritoneum metastasis at 10th month and dead at 28th month
3	*NONO-TFE3*	37/F	5	pT1bNxM0(I)	NSS	13, NED
4	*NONO-TFE3*	46/M	3	pT1aM0N0(I)	NSS	27 NED
5	*NONO-TFE3*	26/M	3.7	pT1aM0N0 (I)	NSS	59, NED
6	*ASPSCR1-TFE3*	22/F	3.9	pT1aM0N1(III)	RN	Bone metastasized at 48th month and dead at 62th month
7	*ASPSCR1-TFE3*	21/M	4	pT1aM0N0(I)	RN	125, NED
8	*ASPSCR1-TFE3*	7/M	3	pT1aM0N0 (I)	RN	145, NED
9	*ASPSCR1-TFE3*	36/F	8.6	pT3cN1M0(III)	RN	Liver metastasized at 2th month and dead at 33th month
10	*ASPSCR1-TFE3*	27/F	6	pT1bN0M0(I)	RN	94, NED
11	*ASPSCR1-TFE3*	25/F	7.1	pT2aN0M0(II)	RN	59, NED
12	*ASPSCR1-TFE3*	38/M	3	pT1aM0N0(I)	RN	66, NED
13	*ASPSCR1-TFE3*	12/F	4.5	pT1bN1M0(III)	RN	19, NED
14	*PRCC-TFE3*	35/M	6	pT1bN0M0(I)	RN	Lunge metastasized at 11th month and dead at 75th month
15	*PRCC-TFE3*	22/F	5	pT1bN0M0(I)	RN	Recued at 12th month and kept stable until 59 months postoperative
16	*PRCC-TFE3*	25/F	3.5	pT1aM0N0(I)	RN	54, NED
17	*PRCC-TFE3*	39/F	4.5	pT1bN0M0(I)	NSS	52, NED
18	*PRCC-TFE3*	45/F	12.4	pT2bN0M0(II)	RN	47, NED
19	*PRCC-TFE3*	30/F	9.5	pT2aN0M0(II)	RN	Peritoneum metastasis at 14th month and dead at 61th month
20	*PRCC-TFE3*	64/M	3	pT1aM0N0(I)	NSS	44, NED
21	*PRCC-TFE3*	26/M	3.7	pT1aM0N0(I)	RN	115, NED

*F* female, *M* male, *NED* no evidence of disease, *NSS* nephron-sparing surgery, *RN* radical nephrectomy.

### Morphology and IHC

**Figure 7:**
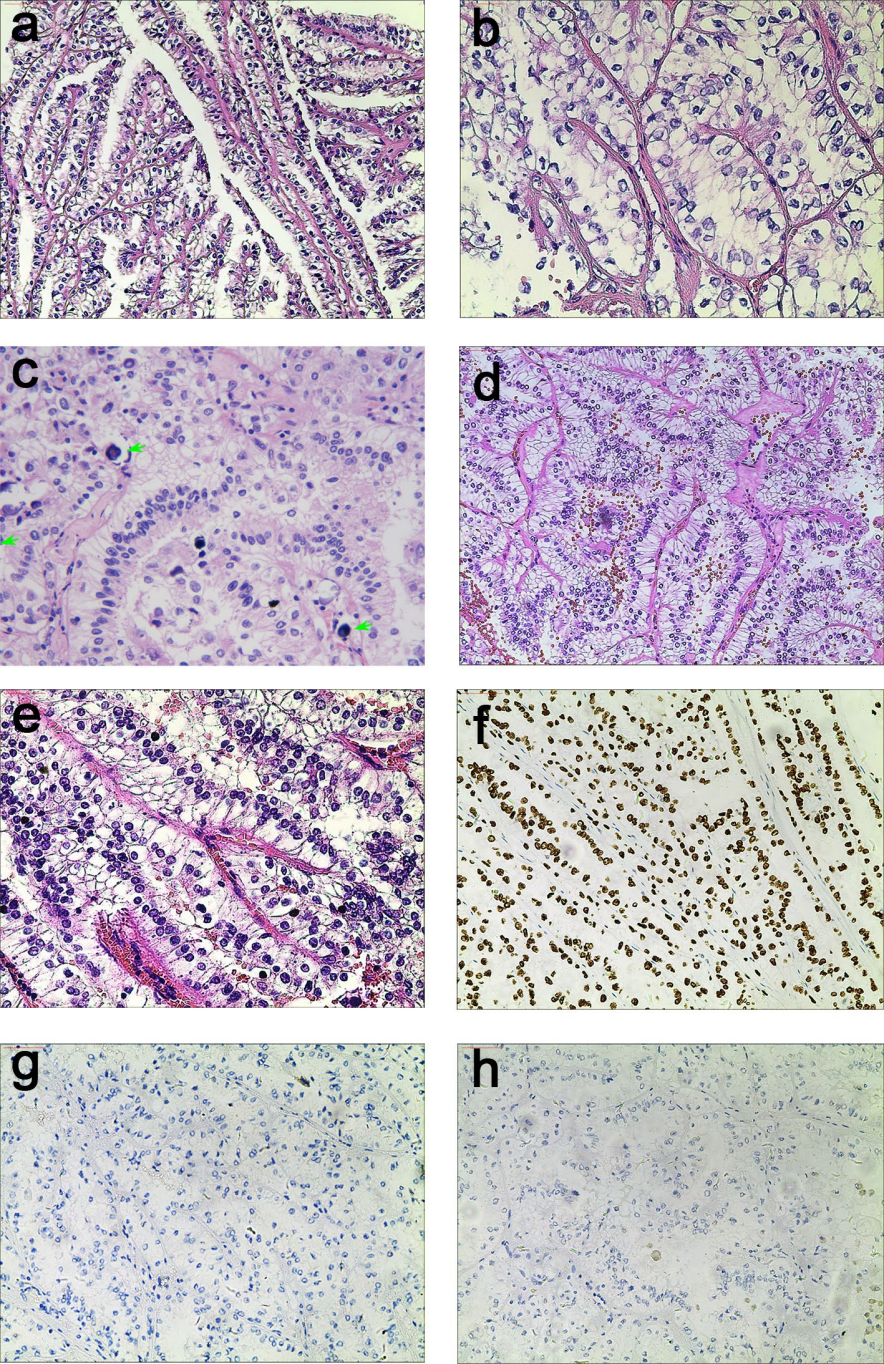
The typical morphologically and IHC feature of *NONO-TFE3* RCC. (**a**–**c**) In cases 1, tumor have predominantly papillary architecture, which features epithelioid cells with clear to finely granular eosinophilic cytoplasm. The neoplastic abundant clear cytoplasm, and nuclei were oriented toward the luminal surface and were round and uniform in shape, resulting in the appearance of secretory endometrioid subnuclear vacuolization. Focal psammoma bodies were also observed (arrows). (**d**, **e**) In cases 4, tumor have predominantly nested to papillary architecture, The neoplastic cells lined with high columnar cells with distinct cell borders, a flocculent eosinophilic cytoplasm, and WHO/ISUP grade 2 nuclei; (**f**) the neoplastic cells demonstrate moderate nuclear labeling for TFE3; (**g**) the neoplastic cells demonstrate negative labeling for CA-IX; (**h**) the neoplastic cells demonstrate negative labeling for CK-7.

All 5 cases displayed a distinctive biphasic pattern in papillary architecture accompanied with sheets of epithelial cells (Fig. 7). The predominantly papillary architecture was lined by columnar cells with clear cytoplasm (Fig. 7b). The nuclear palisading (nuclear palisading means a special pathological phenomenon that the nuclei are arranged as palisade) and subnuclear vacuoles were identified, and nucleoli was not prominent (WHO/ISUP grade 1 or 2; Fig. 7e). Psammoma bodies or calcification were observed in 4 cases (Fig. 7c). Three cases were associated with a predominantly papillary architecture, and the other two showed equal prevalence of papillary architecture and sheets of epithelial cells. Immunohistochemical assays showed that all 5 cases were immunoreactive for TFE3 (Fig. 7f), RCC, CD10, P504S, and Ki-67; but negative for Vim, CA-IX (7 g), and CK7 (Fig. 7h). One out of the five cases had CD117-positive cells, with the other cases being completely negative (Supplementary Table 1).

## Discussion

Xp11.2 translocation RCC is a rare tumor which mainly occurs in children and young adults. Xp11.2 translocation RCC has been reported to have an incidence of 30, 15, and 1.5% in pediatrics, young adults (younger than 45 years), and adults, respectively^16^. The first cases of Xp11.2 translocation RCC involved in t(X; 1) (p11; q21) were reported by de Jong et al. in 1987^17^. However, this rare but aggressive renal tumor did not attract much attention until 2004, when it was classified as a distinctive type of RCC by WHO^18^. In the following decades, distinctive features of Xp11.2 translocation RCC have been increasingly characterized. Nowadays, *ASPSCR1*, *PRCC*, and *SFPQ* are recognized as relatively common gene fusion partners associated with Xp11.2 translocation renal cell carcinoma^8,19^. However, the involvement of *NONO*, *CLTC*, *LUC7L3*, and *RBM10* have only been discussed in case reports^8,20–22^.

FISH is currently the most convenient and effective method for identifying gene alteration by using custom BAC probes on FFPE tissue sections. Utilizing *ASPSCR1-TFE3* and *PRCC-TFE3* dual-fusion FISH assays previously designed by us^5,13^, 8 cases each of *ASPSCR1-TFE3* RCC and *PRCC-TFE3* RCC were identified (Table 1). In the present study, 5 cases of Xp11.2 translocation RCC were found to be positive to the *NONO-TFE3* dual-fusion FISH assay and the diagnosis was eventually confirmed by PCR. Gene sequencing of the PCR products revealed three *NONO-TFE3* fusion points. These are exons 1–7 of *NONO* and exons 6–10 of *TFE3*, exons 1–9 of *NONO* and exons 5–10 of *TFE3,* and exons 1–9 of *NONO* and exons 6–10 of *TFE3.* The former two fusion points have been reported earlier^9^. However, the present study is the first report of the last fusion point. Of the 5 *NONO-TFE3* RCC cases, one (20%) was previously identified as ccRCC owing to equivocal split signals of the *TFE3* gene. A possible remediation is that the possibility of Xp11.2 translocation RCC should not be eliminated if a case is moderate or strong positive to *TFE3* stanning but negative to *TFE3* break-apart assay, and other complementary approaches should be adopted to further clarify and confirm the diagnosis.

In 2017, Xia et al.^9^ introduced a single-fusion FISH assay for the identification of *NONO-TFE3* translocation. Given the adjacent location of *NONO* and *TFE3* genes, only two BAC clones, RP11-934E1 (labeled with 5-fluorescein-dUTP and located centromeric to the *NONO* gene locus) and RP11-416B14 (labeled with 5-ROX-dUTP and located telomeric to the *TFE3* gene locus), were used in their study. Fusion of *TFE3* and *NONO* genes led to overlap of the separated signals. The experimental design of the single-fusion assay ensures rapid analysis of signal patterns (fusion or split). However, other issues could not be suitably clarified by this assay. First, signals consisting of single BAC clones were weak and less obvious, which made data acquisition difficult and increased the possibility of a false-negative diagnosis. Second, it was hard to distinguish target signals from noises, especially for samples with dirty backgrounds, which also increased the difficulty in diagnosis. Third, although single-fusion events represent gene translocation, these may not always balance translocation, which was an important character of Xp11.2 translocation RCC. For example, the fusion of the *ASPSCR1* and *TFE3* genes could occur not only in *ASPSCR1-TFE3* RCC, but also in alveolar soft part sarcoma (ASPS)^23,24^, a stromal tumor involving an unbalanced der (17) t(X; 17) (p11; q25) translocation. As a control, both *TFE3* and *NONO* gene assays in our study consisted of more than 5 BAC clones, which improved signals due to increased signal to noise ratio (SNR). On the other hand, we modified the inter-spot spacing standard as one signal diameter, which was effective in distinguishing fusion signals from split signals. In addition, the dual-fusion assay includes both *NONO-TFE3* fusion and *TFE3-NONO* fusion, and therefore, represents an intuitive and direct response to genetic changes in *NONO-TFE3* RCC. Easy and accurate diagnosis by the dual-fusion assay required information on patient gender, the number of split and fusion signals, and signal size.

By utilizing the *NONO-TFE3* dual-fusion FISH probe, 5 cases of *NONO-TFE3* RCC were diagnosed, accounting for 17.2% of the total Xp11.2 translocation RCC population. In summary, the most distinctive morphological features of the 5 *NONO-TFE3* RCC cases were sheets of epithelial cells and papillary architecture, which were consisted with previous report^8,9^. The cytoplasm was abundantly clear (or flocculent in eosinophilic cells); and the nuclei were rounded and uniform in shape and were oriented toward the luminal surface. Immunohistochemical assays on *NONO-TFE3* RCC showed a strong immunoreactivity (3+++) to TFE3 antibody. According to Green et al.^6,25^, biphasic morphological pattern and strong TFE3 immunoreactivity were important features of *NONO-TFE3* RCC. They proposed that *NONO-TFE3* RCC should be taken into consideration for patients with biphasic morphological pattern and strong TFE3 immunoreactivity, combined with equivocal split signals. In our study, all of these features were verified in all 5 cases of *NONO-TFE3* RCC. Taken together, IHC and break-apart FISH probe could serve as precursor methods, and suspected cases could be further evaluated by the *NONO-TFE3* dual-fusion FISH for final confirmation.

In general, Xp11.2 translocation RCC tends to occur in patients aged between 20 to 50 years, and Xp11.2 translocation RCC is believed to be 2 to 3 times more prevalent in females compared to males^2,8,9,19^. However, our data and previous reports seem to support male predominance in *NONO-TFE3* RCC. Compared with *ASPSCR1-TFE3* RCC and *PRCC-TFE3* RCC (Supplementary Table 2), patients of *NONO-TFE3* RCC were younger and had smaller tumor sizes. In terms of prognosis, *NONO-TFE3* RCC were reported as one of the most moderate subtypes among all Xp11.2 translocation RCC patients^2,9^. Even our data showed that *NONO-TFE3* RCC patients had a longer survival than *ASPSCR1-TFE3* RCC and *PRCC-TFE3* RCC in a short time follow-up study. However, there was a case at the pT1aM0N0 stage, where the patient was diagnosed with peritoneal metastasis at 10 months and dead at 28 months postoperation. The other 4 patients were alive with no evidence of disease progression after their initial resection with a median follow-up of 20.5 months. Given the low number of cases, it’s hard to believe that any of these data are statistically significant. The physiological characteristics of *NONO-TFE3* RCC need further investigation with a larger population and long-term follow-up.

In summary, we reported a newly designed *NONO-TFE3* dual-fusion FISH assay and confirmed the accuracy of this novel probe for detecting *NONO-TFE3* RCC. Five cases of *NONO-TFE3* RCC were identified utilizing this probe and their clinicopathological characteristics were explored. The equivocal split signals make the diagnosis of *NONO-TFE3* RCC difficult. However, this feature could be regarded as a diagnostic marker to provide additional insights to pathologists. In a short-term study, *NONO-TFE3* RCC showed a moderate prognosis compared to that in *ASPSCR1-TFE3* RCC and *PRCC-TFE3* RCC. These findings enhance our understanding of *NONO-TFE3* RCC.

## Materials and methods

### Case selection

The current study was conducted in accordance with the ethical principles of the Helsinki Declaration II. Written informed consent was obtained from both adult patients and legal guardians of pediatric patients before the clinical investigations were performed. This study was approved by the Institutional Review Board of the Nanjing Drum Tower Hospital. 13 confirmed and 5 suspected cases of Xp11.2 translocation RCC, reported from January 2007 to October 2018, were included in this study. In addition, 15 randomly chosen cases each of clear cell renal cell carcinoma (ccRCC) and papillary renal cell carcinoma (pRCC) and UOK 109 cells^10^ (generously gifted by Dr. W. Marston Linehan, National Cancer Institute, Bethesda, MD) were used as controls. All 13 cases of unclassified Xp11.2 translocation RCC were identified by the *TFE3* break-apart FISH probe, but were excluded from *ASPSCR1-TFE3* RCC and *PRCC-TFE3* RCC, by previously reported dual-fusion FISH assays reported by us^5,13^. The 5 suspected cases showed moderate (2+) to strong (3+) immunoblotting response to the TFE3 antibody but equivocal split signals to the *TFE3* probe. All the hematoxylin and eosin (HE) stained tissue sections and IHC sections were reviewed by two experienced pathologists (XHP and SFC). In each case, blocks containing the largest proportion of tumor tissue were selected for subsequent FISH assays. Clinicopathologic features of patients including epidemiological data, clinical manifestations, operation strategies, pathological findings, and prognostic information were recorded and evaluated.

### Reverse transcription–PCR

Total RNA was extracted with the E.Z.N.A. FFPE RNA Kit (Omega Bio-Tek, Guangzhou, China) and complementary cDNA strands were generated using the HiScript Reverse Transcriptase (Vazyme, Nanjing, China). Two primer pairs were designed to detect the fusion transcripts. The PCR primers used were as follows: *NONO* exon 9 (F), ACCTGCCACTATGATGCC; *TFE3* exon 7 (R), TTCTTCTGCCGTTCCTTC; *NONO* exon 7 (F), CGGCAGCAAGAAGAAATG; and *TFE3* exon 8 (R), GGTCACTGGACTTAGGGATG. PCR amplification was performed in a volume of 20 μl under the following conditions: 95 °C for 10 min; followed by 35 cycles of 95 °C for 45 s, 60 °C for 45 s, and 72 °C for 60 s; followed by a final 10-min extension at 72 °C.

PCR products were separated in 1.2% agarose gels and extracted using the TIANgel Midi Purification Kit (Tiangen Biotech, Beijing, China), and subsequent sequence analyses were performed on a BigDye Terminator and ABI Basecaller (Applied Biosystems, Grand Island, NY, USA). The resulting PCR product sequences were compared by using the BLAST algorithm (https://blast.ncbi.nlm.nih.gov/Blast.cgi).

### Dual-fusion dual-color probe design and development

#### Dual-fusion dual-color probe design

Dual-fusion dual-color probe were designed as previously described^5,13^. Suitable BACs for the dual-fusion dual-color probe were selected from https://genome.ucsc.edu/. CTD-3090M11, RP11-485H23, RP11-624G23, CTD-2350P17, and RP11-402P6, labeled with tetramethyl rhodamine-5-dUTP (showing red fluorescence), were located on the long arm of the X chromosome and covered the *NONO* gene. CTD-2311N12, RP11-416B14, CTD-2522M13, CTD-2516D6, CTD-2312C1, CTD-2248C21, and RP11-959H17, labeled with fluorescein-12-dUTP (showing green fluorescence), were located on the short arm of the X chromosome and covered the entire *TFE3* gene (Fig. 1a).

#### FISH experimental procedure

FISH were performed as previously described^9,11^. 3 mm thick sections from formalin-fixed, paraffin-embedded (FFPE) tumor tissue blocks were first heated overnight in an incubator maintained at 65 °C. Then, sections were deparaffinized by treatment with xylene for 30 min, followed by washing with absolute ethanol for 10 min. Sections were rehydrated in 100, 90, and 70% ethanol and double distilled water, in turn, for 3 min. This was followed by boiling these sections in sterilized purified water for 20 min and subsequent air drying. Sections were then digested with 10 μl pepsin (4 mg/mL, 0.02 M HCl; Sigma-Aldrich, Beijing, China) at 37.5 °C for 5 to 10 min and washed with 2X saline–sodium citrate (SSC) for 5 min. Subsequently, these sections were dehydrated in 70, 90, and 100% ethanol, in turn, for 3 min and air dried. Hybridization was performed with the newly designed FISH probe, denatured at 85 °C for 5 min, and incubated overnight (10–18 h) at 37 °C under lucifugal conditions. The sections were then immersed in 2X SSC for 10 min; and in 0.1% NP-40 and 2X SSC for 5 min at 37 °C. Then, the sections were immersed in 70% ethanol for 3 min and air dried. The nuclei were counterstained with 10 μl of 4′,6-diamidino-2-phenylindole (DAPI). After hybridization, all slides were stored in the dark.

#### FISH evaluation

Evaluation of FISH results was conducted with an Olympus BX51 fluorescence microscope (Olympus, Tokyo, Japan) fitted with 3 filters (DAPI/FITC/Texas Red) and the FISH analysis software (Imstar, Paris, France). The FISH assay is effective only when clear FISH signals are observed in > 100 nonoverlapping nuclei.

## Supplementary information

Supplementary Tables.
