# Supplementary material for: The suitability of NONO-TFE3 dual-fusion FISH assay as a diagnostic tool for NONO-TFE3 renal cell carcinoma
Source: Sci Rep. 2020 Oct 1;10:16361. doi: 10.1038/s41598-020-73309-4 (PMC7530984; doi:10.1038/s41598-020-73309-4)
Supplement: Supplementary file 1 — Supplementary Tables. [file 41598_2020_73309_MOESM1_ESM.pdf]

The suitability of *NONO-TFE3* dual-fusion FISH assay as a diagnostic tool for *NONO-TFE3* renal cell carcinoma

Ning Liu[1], Wei Guo[2,3], Qiancheng Shi[1], Wenyuan Zhuang[1], Xiaohong Pu[4], Shaoyu Chen[5], Feng Qu[1], Linfeng Xu[1], Xiaozhi Zhao[1], Xiaogong Li[1], Gutian Zhang[1], Hongqian Guo[1], Weidong Gan[1,2,+,*], Dongmei Li[6,7,+,*]

Supplementary Table 1. Immunohistochemical findings for *NONO-TFE3* renal cell carcinomas.

Case	TFE3	TFEB	RCC	CD10	CD117	P504S	Ki-67	Vim	CA-IX	CK-7
1	+++	-	+++	+++	-	+++	1%	-	-	-
2	+++	-	+++	+++	-	++	3%	-	-	-
3	+++	+	+	+	-	+	4%	-	-	-
4	+++	-	++	++	+	+	10%	-	-	-
5	+++	-	+++	++	-	+	2%	-	-	-

Supplementary Table 2. Comparison of three genotypes of Xp11.2 translocation RCC.

Fusion type	*ASPSCR1-TFE3*	*PRCC-TFE3*	*NONO-TFE3*
Median age	23.5 ys (7 ys to 38 ys)	32.5 ys (22 ys to 64 ys)	41.2 ys (26 ys to 55 ys)
Gender ratio (Female to male)	5:3	5:3	2:3
Median Tumor size	4.3 cm (3-8.6 cm)	4.8 cm (3-12.4 cm)	3.6 cm (3-5 cm)
Pathology features	Alveolar or pseudopapillary architecture; extensive psammomatous calcification; Positive to TFE3 IHC; Positive to *TFE3* spilt FISH assays; Positive to *ASPSCR1-TFE3* dual-fusion FISH assays	Alveolar or pseudopapillary architecture; less psammomatous calcification; Positive to TFE3 IHC; Positive to *TFE3* spilt FISH assays; Positive to *PRCC-TFE3* dual-fusion FISH assays	Biphasic pattern; columnar cells with clear cytoplasm; Positive to TFE3 IHC; Equivocal *TFE3* spilt FISH assays; Positive to *NONO-TFE3* dual-fusion FISH assays
1 years PFS	87.5%	75.0%	80%
3 years PFS	72.9%	62.5%	80%

PFS: progression free survival.
